# Differences in Long-Term Heart Rate Variability between Subjects with and without Metabolic Syndrome: A Systematic Review and Meta-Analysis

**DOI:** 10.3390/jcdd10050203

**Published:** 2023-05-09

**Authors:** Johan E. Ortiz-Guzmán, Sara Mollà-Casanova, Óscar J. Arias-Mutis, Alexandra Bizy, Conrado Calvo, Antonio Alberola, Francisco J. Chorro, Manuel Zarzoso

**Affiliations:** 1 Department of Physiology, Universitat de València, 46010 Valencia, Spain; jorguz@alumni.uv.es (J.E.O.-G.); conrado.calvo@uv.es (C.C.); antonio.alberola@uv.es (A.A.); 2 UBIC Research Group, Department of Physiotherapy, Universitat de València, 46010 Valencia, Spain; sara.molla@uv.es; 3 Department of Biomedical Sciences, CEU Cardenal Herrera, 46115 Valencia, Spain; oscarariasphd@gmail.com (Ó.J.A.-M.); alexandra.bizy@uchceu.es (A.B.); 4 Health Research Institute—Instituto de Investigación Sanitaria del Hospital Clínico Universitario de Valencia (INCLIVA), Department of Medicine, Universitat de València, 46010 Valencia, Spain; francisco.j.chorro@uv.es; 5 Centro de Investigación Biomédica en Red de Enfermedades Cardiovasculares (CIBER-CV), 28029 Madrid, Spain; 6 Department of Physiotherapy, Universitat de València, 46010 Valencia, Spain

**Keywords:** long-term heart rate variability, metabolic syndrome, meta-analysis

## Abstract

Background: Our aim was to determine the impact that metabolic syndrome (MS) produces in long-term heart rate variability (HRV), quantitatively synthesizing the results of published studies to characterize the cardiac autonomic dysfunction in MS. Methods: We searched electronic databases for original research works with long-term HRV recordings (24 h) that compared people with MS (MS+) versus healthy people as a control group (MS−). This systematic review and meta-analysis (MA) was performed according to PRISMA guidelines and registered at PROSPERO (CRD42022358975). Results: A total of 13 articles were included in the qualitative synthesis, and 7 of them met the required criteria to be included in the MA. SDNN (−0.33 [−0.57, 0.09], *p* = 0.008), LF (−0.32 [−0.41, −0.23], *p* < 0.00001), VLF (−0.21 [−0.31, −0.10], *p* = 0.0001) and TP (−0.20 [−0.33, −0.07], *p* = 0.002) decreased in patients with MS. The rMSSD (*p* = 0.41), HF (*p* = 0.06) and LF/HF ratio (*p* = 0.64) were not modified. Conclusions: In long-term recordings (24 h), SDNN, LF, VLF and TP were consistently decreased in patients with MS. Other parameters that could be included in the quantitative analysis were not modified in MS+ patients (rMSSD, HF, ratio LF/HF). Regarding non-linear analyses, the results are not conclusive due to the low number of datasets found, which prevented us from conducting an MA.

## 1. Introduction

The clustering of dyslipidemia, hypertension and hyperglycemia was initially called the Syndrome X or insulin resistance syndrome by Reaven, and it was linked to the development of cardiovascular disease (CVD), developed on the basis of insulin resistance [1]. At the beginning of the 21st century, the National Cholesterol Education Program’s Adult Treatment Panel III (ATP III) called it metabolic syndrome (MS), since insulin resistance was not the only cause of the association between these risk factors [2]. In the same way, the ATP III concluded that the main clinical identification of MS should be abdominal obesity, dyslipidemia, blood pressure and fasting glucose [2,3].

As the global prevalence of MS is rising to pandemic proportions, it is considered nowadays one of the main Public Health challenges [4,5], and although the underlying pathophysiological mechanisms are not yet fully elucidated, there is enough scientific evidence indicating that dysfunction in the control exerted by the autonomic nervous system (ANS) on cardiovascular behavior is one of the main mechanisms that would explain the increased risk of morbidity and mortality associated with MS [6,7]. Indeed, Endukuru et al. found altered baroreflex sensitivity, higher resting heart rate and reduced heart rate variability (HRV) values in patients with MS, all of which are related to cardiac autonomic dysfunction [8]. Likewise, Li et al. studied the association between MS and cardiovascular autonomic function in 2119 subjects finding that as the MS score increases, the HRV spectral variables (TP, LF, HF) decrease, a relationship that is maintained even after adjusting for age, gender, serum creatinine and uric acid [9]. In the same way, Azulay et al., after studying the relationship between the number of components of MS and the behavior of HRV in a sample of 7880 subjects, concluded that autonomic dysfunction appears even in earlier stages, and that a correct screening could control its progressions [10].

Given the potential contribution of ANS dysfunction in the development of MS and its major cardiovascular complications, HRV has been well recognized for its predictive power [11,12]. HRV provides a noninvasive tool, based on the analysis of fluctuations between heartbeats, and it is generated by dynamic processes that result mainly from the interaction between the ANS and cardiovascular function [13]. HRV can be measured in short (±5 min) or long (24-h) periods of recording [14]; even ultra-short periods (<1 min) have also shown significant prognostic value [15]. Nevertheless, long-term recordings have been considered by many authors as the gold standard for diagnosis and clinical application, since their measurements offer greater predictive power than the results obtained in short-term recordings [13,16].

Despite the existence of sufficient scientific evidence that confirms the disbalance of the autonomic control over cardiovascular activity associated with MS, there are still no studies that have quantitatively characterized, using the meta-analysis (MA), the modifications of HRV by means of long-term recordings. For this reason, we have conducted this systematic review and MA to characterize the cardiac autonomic dysfunction present in patients with MS, in order to identify the most frequently reported explanatory variables.

## 2. Materials and Methods

The study was registered at the International Prospective Register of Systematic Reviews (PROSPERO) at https://www.crd.york.ac.uk/prospero/ (accessed on 19 October 2022 (CRD42022358975)).

### 2.1. Search Strategy

A systematic review of the scientific literature was performed following the PRISMA (Preferred Reporting Items for Systematic Reviews and Meta-analyses) guidelines [17]. The articles were searched in the specialized databases of the Web of Science, Scopus and the US National Library of Medicine (PubMed), where MeSH terms were used to delimit the search of the reviewed topic. Within these databases, the main term used was “metabolic syndrome” linked to “heart rate variability”, “cardiac autonomic control”, “cardiac autonomic function” or “cardiac autonomic modulation”. In the construction of the search equations, the Boolean characters AND and OR were used, and the search was limited to the title and abstract fields (Table 1). The search for articles was carried out between 25 June 2022 and 1 September 2022.

### 2.2. Study Inclusion/Exclusion Criteria

Studies were included in the systematic review and metanalysis if they were (i) original works; (ii) performed on humans; (iii) with long-term HRV recordings (24 h); (iv) that compare people with MS (MS+) versus healthy people (i.e., without MS) as a control group (MS−); (v) that include an assessment of time or frequency domains or non-linear analyses; and (vi) written in English. No filter was applied by the publication date of the articles. A detailed description of the variables can be found in Appendix A.

Exclusion criteria included (i) systematic reviews of the literature and/or MA, (ii) bibliographic reviews, (iii) letters to the editor or (iv) conference communications.

### 2.3. Quality Assessment

In order to evaluate the quality of the studies, a table was built to collect general information about the publication (title, authors, journal and year of publication, objective), characteristics of the population studied (sample size by groups, gender, age, weight, height, Body Mass Index -BMI-), HRV measurement (length of recording time and hour of the register, ventilation control, previous fasting, body position, variables measured in time and dominant frequency and in non-linear analysis), criteria for MS diagnostic and main results.

The methodological quality evaluation of the studies was carried out following the recommendations made by Law et al. [18,19] for the review of quantitative articles. According to this proposal, each article is evaluated using 16 items. The result of the evaluation of each article is expressed as a percentage, which is calculated by adding the total number of items contained and dividing this result by 16. The articles were classified as “low methodological quality” when their result was less than or equal to 50%; “good methodological quality” when they reached a score between 51 and 75%; and “excellent methodological quality” with a score of 76% or more [19].

### 2.4. Data Extraction

The Endnote^®^ bibliographic manager was used to manage all the records retrieved during the review. A qualitative synthesis was performed using the 13 studies obtained in the systematic review. The information was organized in a table using Microsoft Excel (2019), in which the quality of studies, participants’ demographic data and assessment outcomes were detailed. Moreover, the outcomes’ result behavior was described in another Microsoft Excel (2019) table.

After an accurate qualitative synthesis, a quantitative synthesis was performed. For the assessment of the study heterogeneity, the clinical and methodological diversity of the studies included was assessed to determine if an MA was appropriate. For that purpose, the I² statistical test was used. As recommended by the Cochrane Handbook [20], a heterogeneity range of 0–40% might not be important; 30–60% may represent moderate heterogeneity; 50–90% may represent substantial heterogeneity; and 75–100% represents considerable heterogeneity [20].

After that, to analyze the differences between the groups on the variables, MA using RevMan 5.4 was conducted when 3 or more articles measured the same outcome. Before pooling data, comparisons were grouped as MS+ versus MS−. To report the differences between groups, Cohen’s d and the 95% Confidence Interval (CI) were used as indicators of the standard mean difference (SMD) (when different outcome measures were combined), mean difference (MD) (when different outcome measures were not combined) and significance.

## 3. Results

### 3.1. Identification of Studies

**Figure 1:**
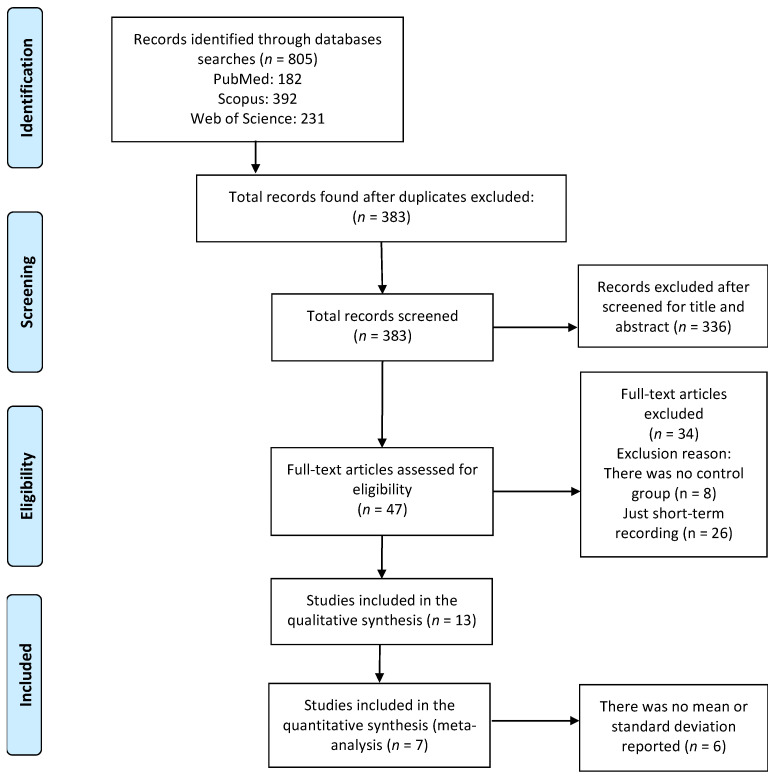
Preferred reporting items for systematic reviews and meta-analyses flow diagram illustrating selection of studies.

We found 805 articles in the initial review (182 in PUBMED, 392 in Scopus and 231 in Web of Science), of which 422 were excluded because they were repeated in the selected databases. The remaining 383 records were reviewed (title and abstract) and a total of 336 studies were excluded (201 for not measuring HRV, 61 for analyzing only 1 component of the MS, 48 for not being original articles and 26 for being studies in animal models). Subsequently, 47 full-text papers were retrieved, which were reviewed to ensure that they met the inclusion and exclusion criteria. Of these, 8 were excluded from the final analysis for not making comparisons between people with MS+ vs. MS− and 26 for using short-term recordings (≤5 min). No study was excluded for being assessed as “low methodological quality”; thus, 13 articles were included in the final qualitative synthesis (Figure 1). Finally, seven studies met the required criteria to be included in the quantitative synthesis (MA).

### 3.2. Quality Assessment

As a result of the methodological evaluation, there were 11 articles (85%) valued as “excellent methodological quality”; 2 (15%) scored as “good methodological quality”; and no reviewed work was in “low methodological quality”. On average, the methodological quality of all reviewed studies was 88% (“excellent methodological quality”).

### 3.3. Study and Patient Characteristics

A summary of the studies’ characteristics included in this review is shown in Table 2. No study was published before the year 2000, 5 were published between 2006 and 2010, and 8 between 2013 and 2020. The population studied was from: (a) North America (*n* = 3), all of them from United States [21,22,23]; (b) Europe (*n* = 7), where two of them were from Poland [24,25] and one from France [26], Serbia [27], Germany [28], Turkey [29] and Lithuania [30]; and (c) Asia (*n* = 3), retrieving one from Japan [31], Taiwan [11] and Korea [32].

Regarding sex, 2 studies included only men [22,24], and 11 studies were carried out in both men and women [11,21,23,25,26,27,28,29,30,31,32]. No analyses between groups (men vs. women) were made in any study. With respect to age, just 1 study included people under 30 years old [28]; 5 papers included people between 34 and 60 years old [22,23,24,25,27]; and 7 papers evaluated the population over 43 years old [11,21,26,29,30,31,32].

Regarding MS diagnosis criteria, only one study reported the average number of factors that contribute to the presentation of MS in their participants [29], and one more reported the percentage of participants that had each component [30]. Moreover, all the studies reported the criteria used for the diagnosis of MS (Table 2). In total, the 13 studies included in this review have a sample of 5987 participants, 2334 (39%) being patients with MS.

According to the criteria described for the quantitative analysis, the R-R interval, SDNNi, SDANN, pNN20, pNN50, ULF and non-linear variables were not analyzed quantitatively (highlighted in bold in Table 2). All other linear variables, both in the time domain and in the frequency domain, were included in the quantitative analysis (i.e., SDNN, rMSSD, HF, LF, LF/HF, VLF and TP).

### 3.4. Time Domain Analysis Outcomes

Of the 13 studies included in the review, 9 of them analyzed time domain variables, 8 in mixed group and 1 in men, and just 1 study performed analyses in people under 40 years old. A summary of the main findings is presented in Table 3.

For the R-R interval, no changes were reported for mixed groups older than 40 years [11,21,29,30]. Regarding pNN50, two studies reported decreased values in mixed groups with MS+ [25,29] or in men [24], and two additional studies did not find significant changes for this variable [11,30]. For the pNN20, only one study looked for changes in this parameter, finding lower values in persons with MS+ [11]. The variables SDANN [21,24,29,32] and SDNNi [24,29,30,32] were found to decrease for the MS+ group, except for Slušnienė et al. [30] who did not find changes in SDANN.

**Figure 2:**
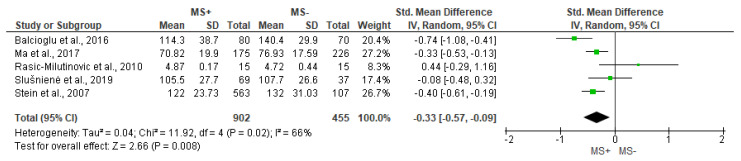
Comparison of the effects of metabolic syndrome (MS+) versus control (MS−) group on SDNN using forest plot. Each study is represented by a green dot at the point estimate of intervention effect with a horizontal line extending either side of the dot. The area of the dot indicates the weight assigned to that study in the meta-analysis while the horizontal line depicts the confidence interval (95% level of confidence). Balcioglu et al., 2016 [29]; Rasic-Milutionovic et al., 2010 [27]; Ma et al., 2017 [11]; Slušnienė et al., 2019 [30]; Stein et al., 2007 [21].

The SDNN outcome is studied by all works reviewed and, thus, is included in the MA. Figure 2 shows significant differences between groups for SDNN outcome, being lower in people with MS+ (SMD = −0.33 [95% IC = −0.57, −0.09], *p* = 0.008), with substantial heterogeneity between reports (I^2^ = 66%).

**Figure 3:**
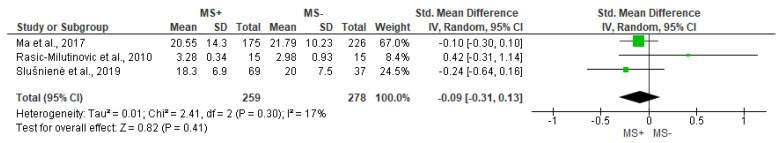
Comparison of the effects of metabolic syndrome (MS+) versus control (MS−) group on rMSSD using forest plot. Each study is represented by a green dot at the point estimate of intervention effect with a horizontal line extending either side of the dot. The area of the dot indicates the weight assigned to that study in the meta-analysis while the horizontal line depicts the confidence interval (95% level of confidence). Ma et al., 2017 [11]; Rasic-Milutinovic et al., 2010 [27]; Slušnienė et al., 2019 [30].

On the other hand, no significant differences between groups were found for rMSSD in the MA (*p* = 0.41), with small heterogeneity between studies (I^2^ = 17%, Figure 3).

### 3.5. Frequency Domain Analysis Outcomes

In total, nine studies performed analyses of the behavior of the spectral parameters of HRV. A summary of the main findings is presented in Table 4.

**Figure 4:**
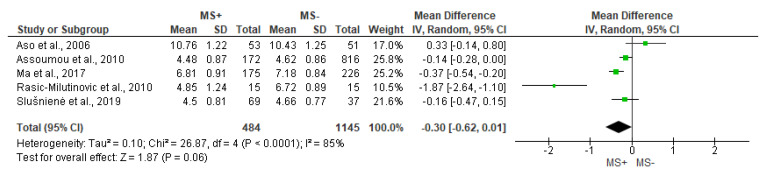
Comparison of the effects of metabolic syndrome (MS+) versus control (MS−) group on HF using forest plot. Each study is represented by a green dot at the point estimate of intervention effect with a horizontal line extending either side of the dot. The area of the dot indicates the weight assigned to that study in the meta-analysis while the horizontal line depicts the confidence interval (95% level of confidence). Aso et al., 2006 [31]; Assoumou et al., 2010 [26]; Ma et al., 2017 [11]; Rasic-Milutinovic et al., 2010 [27]; Slušnienė et al., 2019 [30].

Regarding the HF, and with substantial heterogeneity (I^2^ = 85%), the analysis did not report significant differences between groups (*p* = 0.06), but the test for the overall effect was almost significant (Figure 4).

**Figure 5:**
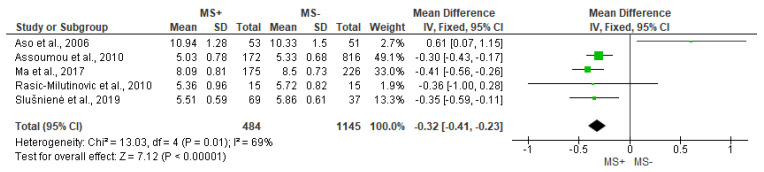
Comparison of the effects of metabolic syndrome (MS+) versus control (MS−) group on LF using forest plot. Each study is represented by a green dot at the point estimate of intervention effect with a horizontal line extending either side of the dot. The area of the dot indicates the weight assigned to that study in the meta-analysis while the horizontal line depicts the confidence interval (95% level of confidence). Aso et al., 2006 [31]; Assoumou et al., 2010 [26]; Ma et al., 2017 [11]; Rasic-Milutinovic et al., 2010 [27]; Slušnienė et al., 2019 [30].

Figure 5 depicts the analysis of LF. Significantly lower values of LF for the MS+ group compared to MS− were found (MD = −0.32 [95% IC = −0.41, −0.23], *p* < 0.00001), albeit the results show substantial heterogeneity (I^2^ = 69%).

**Figure 6:**
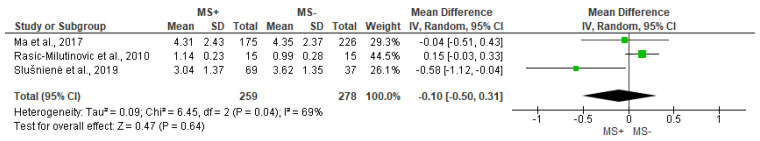
Comparison of the effects of metabolic syndrome (MS+) versus control (MS−) group on LF/HF using forest plot. Each study is represented by a green dot at the point estimate of intervention effect with a horizontal line extending either side of the dot. The area of the dot indicates the weight assigned to that study in the meta-analysis while the horizontal line depicts the confidence interval (95% level of confidence). Ma et al., 2017 [11]; Rasic-Milutinovic et al., 2010 [27]; Slušnienė et al., 2019 [30].

Regarding LF/HF, we found substantial heterogeneity (I^2^ = 69%) between studies, and no significant differences (*p* = 0.64) were found between groups (Figure 6).

**Figure 7:**
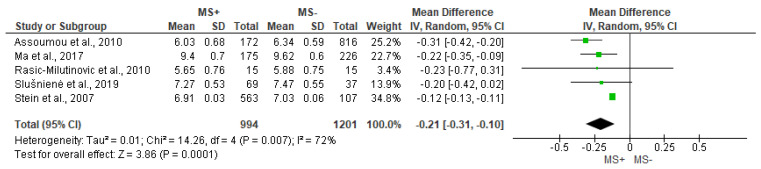
Comparison of the effects of metabolic syndrome (MS+) versus control (MS−) group on VLF using forest plot. Each study is represented by a green dot at the point estimate of intervention effect with a horizontal line extending either side of the dot. The area of the dot indicates the weight assigned to that study in the meta-analysis while the horizontal line depicts the confidence interval (95% level of confidence). Assoumou et al., 2010 [26]; Ma et al., 2017 [11]; Rasic-Milutinovic et al., 2010 [27]; Slušnienė et al., 2019 [30]; Stein et al., 2007 [21].

**Figure 8:**
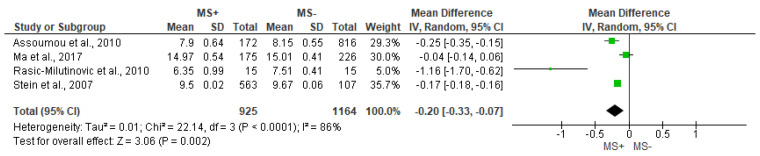
Comparison of the effects of metabolic syndrome (MS+) versus control (MS−) group on TP using forest plot. Each study is represented by a green dot at the point estimate of intervention effect with a horizontal line extending either side of the dot. The area of the dot indicates the weight assigned to that study in the meta-analysis while the horizontal line depicts the confidence interval (95% level of confidence). Assoumou et al., 2010 [26]; Ma et al., 2017 [11]; Rasic-Milutinovic et al., 2010 [27]; Stein et al., 2007 [21].

The analysis of VLF (Figure 7) and TP (Figure 8) showed significant differences between MS+ and MS− groups (MD = −0.21 [−0.31, −0.10], *p* = 0.0001 and MD = −0.20 [−0.33, −0.07], *p* = 0.002, respectively). In both cases, there was substantial heterogeneity among studies (VLF I^2^ = 72% and TP I^2^ = 86%).

For the ULF, the outcomes reported show a decrease in patients with MS+ in mixed groups [21,26] and in men [22,24], but the MA could not be performed.

### 3.6. Non-Linear Analysis Outcomes

Regarding the non-linear analysis, only four studies included these variables, and their results are not consistent (Table 5). For the Poincaré Plot, Slušnienė et al. [30] found no significant differences between groups for SD1 and SD2. Similarly, Ma et al. [11] did not find any changes in SD1 but reported a decrease in SD2 for the MS+ group. Conversely, Assoumou et al. [26] concluded that there were no significant differences in α1 (detrended fluctuations analysis) between groups, but Stein et al. [21] reported a decrease in this parameter in patients with MS. Finally, Ma et al. found no significant changes in entropy (multiscale analysis) [11].

## 4. Discussion

We conducted this systematic review and MA to determine the impact that MS produces in long-term HRV, characterizing the cardiac autonomic dysfunction induced by this pathological condition with a qualitative analysis of the most frequently reported variables and a subsequent quantitative analysis to identify the main explanatory variables. A total of 13 moderate-high quality studies were reviewed, 7 of which were included in the MA.

The main findings of the MA were the following: (1) patients with MS exhibited changes in long-term HRV in the time-domain analyses, showing lower SDNN values but no modifications in rMSSD; (2) regarding the frequency-domain analyses, patients with MS present lower values in LF, VLF and TP, except for the HF and the ratio LF/HF, which were not different; (3) we could not perform subgroup analyses using sex as a factor, since only two studies reported dataset in men and no studies reported any data in women; and (4) studies reporting non-linear analyses were scarce, and thus, the evidence presented was limited.

Regarding time domain parameters, SDNN is modulated by both sympathetic (SNS) and parasympathetic (PNS) nervous system activity, and it has been widely related to spectral variables such as ULF, VLF, LF and TP [13,14]. The quantitative analysis using MA showed significantly lower values in SDNN in the MS+ group, albeit with substantial heterogeneity. This parameter is considered the “gold standard” for cardiac risk stratification of patients over a 24 h period [14] and suggests an important imbalance in cardiac autonomic control in this population. Even though the prognostic value of this parameter has not been determined in MS, it has been shown that in patients after acute myocardial infarction, values over 100 ms were associated with a 5.3 times lower risk of mortality [34]. The rMSSD was also analyzed in the MA and provides information about the activity of the PNS. The rMSSD is a variable that reflects the beat-to-beat variation, since its calculation is based on the analysis of the differences of successive N-N intervals and has an important prognostic value [13,35]. This parameter is also related to the HF band [14] and the SD1 of the non-linear analysis using Poincaré Plot [13,36]. In a recent MA performed to identify predictors of mortality using HRV, Jarczok et al. concluded that rMSSD in short-term recording periods (5 min) are more useful for risk stratification of morbidity and/or mortality from different causes, while those obtained in long recording periods (24 h) are more important for analyzing additional information, such as stress factors during work shifts, ambient temperature, circadian control and other factors. [37]. However, the MA found no statistically significant differences over 24 h-long recordings, in this case with small heterogeneity among studies.

Frequency domain measurements are used to separate HRV into its different components (ULF, VLF, LF and HF rhythms). The MA showed a significant decrease in the LF component of the frequency domain in patients with MS. This frequency band is produced by both sympathetic and parasympathetic branches of the ANS, as well as being influenced by the arterial pressure regulatory mechanisms, via baroreceptors [14,38]. Although the interpretation of this parameter could be difficult given the different sources that modulate it, several studies have already highlighted that LF has an inverse association with the components of MS, as well as with MS in general [22,26,27], pointing towards a lower HRV in this population. In the same line, the result of this MA showed a significant decrease in VLF in the MS+ group. Regarding its physiological significance, it has been suggested that the modulation exerted by the ANS on cardiac activity is not primarily responsible for the frequencies found in the lower bands of the spectrum, such as ULF and VLF (frequencies < 0.04 Hz), and that the analysis of these frequencies, especially the VLF, provides information about the long-term regulation of cardiac activity, being the main generator of the spectral power found in the interval between beats [39,40]. Therefore, current evidence suggests that this signal comes from an intrinsic property of the heart known as the “coupled-clock pace-maker cell system”, and that it is responsible for both the generation of the action potential in pacemaker cells, as well as its rhythmicity [40,41]. Different studies have concluded that low values in VLF are related to worse clinical outcomes in different pathological conditions, providing even greater prognostic value than LF or HF [13,42,43,44]. Likewise, an inverse relationship between VLF and proinflammatory states has been reported in several studies [45,46,47], which could be related with MS, since it is considered a condition which coexists with a low-grade chronic inflammatory processes [47].

On the other hand, HF reflects parasympathetic activity [14], and it is also called the “respiratory band” due to its dependence on the respiratory cycle, decreasing during inhalation and increasing during exhalation [13]. Furthermore, this parameter is highly correlated with the pNN50 and rMSSD time-domain measures [48]. Even though HF has a high prognostic value for cardiovascular risk and mortality [49], the MA showed no significant differences between MS+ and MS−. Of note, the test for overall effect was almost significant (*p* = 0.06), but the high heterogeneity found (I^2^ = 85%) might be responsible for this result. The LF/HF ratio of the spectral analysis was also analyzed in the MA, but no statistically significant differences were found between MS+ and MS−.

Finally, another frequency domain parameter that could be included in the MA was the TP, which was found to be significantly decreased in MS+. TP is representative of the behavior of all the different bands of the total spectrum, and several studies have associated low TP values with a higher incidence of cardiovascular risk factors, as well as for the development of diabetes mellitus [24,50].

Regarding the non-linear analysis of HRV, it was not possible to perform a quantitative analysis due to the low number of datasets found in the systematic review. Although there is still some uncertainty about the physiological significance of nonlinear parameters for HRV analysis [14], these measures have shown to be useful for the assessment of cardiovascular risk and sudden cardiac death [13] and are a well-recognized way to distinguish between physiological and pathological conditions [11]. The analyses of the complexity (entropy) and the fractal component of the signal (detrended fluctuations analysis) provide very important information for the analysis of the complex mechanisms of HRV regulation. Even a qualitative analysis showed that the results of the studies reviewed are not conclusive in respect of differences in the behavior of HRV in people with MS. Thus, more research focused on the use of non-linear analyses in MS would be necessary.

The findings of this systematic review and MA are important as they address, not only qualitatively but also quantitatively, the characterization of the cardiac autonomic dysfunction present in patients with MS, which enabled us to identify the most frequently reported explanatory parameters. However, we did not take into account some covariates such as (1) morphological measurements (body mass index, waist-to-height ratio or waist circumference) which are related to HRV; (2) the amount of physical activity given its capacity to modify HRV (it was barely reported); (3) methodological aspects related to signal acquisition and processing such as the method used for frequency domain parameters estimation (Fast-Fourier vs. Autoregressive), sampling frequency or post-processing of the data, which were beyond the scope of our work; and (4) respiratory rate was not reported in the studies included in this MA. As already shown, obesity is usually accompanied by hyperleptinemia, which leads to a stage of systemic leptin resistance [51]. In turn, leptin has been closely related to poorer respiratory control and a mismatch in response to hypercapnic states [52]. This could suggest that, in people with MS, especially those with abdominal obesity and dyslipidemia, ventilatory control could be compromised, and differences in respiratory rate might induce changes in HRV. Thus, it would be advised that future studies take into account the respiratory rate, if possible, due to its influence on HRV.

## 5. Conclusions

In conclusion, this systematic review of the literature and MA showed that, in long-term recordings (24 h), patients with MS consistently showed lower values in SDNN, LF, VLF and TP. Other parameters that could be included in the quantitative analysis were not different between MS^+^ and MS- patients (rMSSD, HF, ratio LF/HF). Regarding non-linear analyses, the results are not conclusive due to the low number of datasets found, which prevented us from conducting an MA.

## Figures and Tables

**Table 1:** Search strategy.

Database	Search Equation
PubMed	((“heart rate variability” [Title/Abstract] OR “autonomic control” [Title/Abstract] OR “HRV” [Title/Abstract] OR “cardiac autonomic control” [Title/Abstract] OR “cardiac autonomic function” [Title/Abstract] OR “cardiac autonomic modulation” [Title/Abstract]) AND (“metabolic syndrome” [Title/Abstract]))
Web of Science	(“heart rate variability” OR “autonomic control” OR “HRV” OR “cardiac autonomic control” OR “cardiac autonomic function” OR “cardiac autonomic modulation”) AND (“metabolic syndrome”)
Scopus	(TITLE-ABS-KEY (“metabolic syndrome”) AND TITLE-ABS-KEY (“heart rate variability”) O TITLE-ABS-KEY (“autonomic control”) OR TITLE-ABS-KEY (“HRV”) OR TITLE-ABS-KEY (“cardiac autonomic control”) OR TITLE-ABS-KEY (“cardiac autonomic function”) OR TITLE-ABS-KEY (“cardiac autonomic modulation”))

**Table 2:** Summary of the studies’ characteristics included in the review.

Reference	Methodological Evaluation (%)	*n*	Age (Years)	Sex (F/M)	MS Definition	Analyzed Variables		
						Time	Frequency	Non-Linear
Aso et al., 2006 [31]	94%	104	43–70	Both (48/52)	NCEP ATP III	No	HF, LF, LF/HF	No
Stein et al., 2007 [21]	75%	899	67–76	Both (56/44)	NCEP ATP III	SDNN, **SDANN**, **SDNNi**, rMSSD, **pNN50**	HF, LF, TP, VLF, **ULF**	DFA-1
Gehi et al., 2009 [22]	88%	288	50–57	Men (0/100)	AHA y NHLBI	No	HF, LF, TP, VLF, **ULF**	No
Assoumou et al., 2010 [26]	94%	1010	64–66	Both (60/40)	NCEP-ATP III	No	TP, HF, LF, VLF, **ULF**, LF/HF	No
Rasic-Milutinovic et al., 2010 [27]	88%	47	50–60	Both (60/40)	NCEP ATP III	SDNN, rMSSD	HF, LF, LF/HF, TP, VLF	No
Poliwczak et al., 2013 [24]	94%	80	50–55	Men (0/100)	IDF	SDNN, **SDANN**, **SDNNi**, rMSSD, **pNN50**	HF, LF, LF/HF, TP, VLF, **ULF**	No
Jarczok et al., 2013 [28]	88%	2441	18–67	Both (24/76)	IC	SDNN, rMSSD	HF, LF, LF/HF	No
Wulsin et al., 2016 [23]	94%	1143	40–57	Both (57/43)	IC	SDNN, rMSSD	No	No
Yoo et al., 2016 [32]	94%	1200	50–60	Both (60/40)	NCEP ATP III	SDNN, **SDANN**, rMSSD	No	No
Balcioglu et al., 2016 [29]	94%	150	48–74	Both (65/35)	NCEP ATP III	SDNN, **SDANN**, **SDNNi**, rMSSD, **pNN50**	No	No
Ma et al., 2017 [11]	94%	401	46–64	Both (41/59)	NCEP ATP III	SDNN, rMSSD, **pNN50**, **pNN20**	HF, LF, LF/HF, TP, VLF	SD1, SD2, SD1/SD2, Multiscale entropy
Slušnienė et al., 2019 [30]	81%	106	50–55	Both (49/51)	NCEP ATP III	SDNN, **SDANN**, **SDNNi**, rMSSD, **pNN50**	HF, LF, LF/HF, VLF	No
MacIorowska et al., 2020 [25]	69%	118	34–58	Both (32/86)	IDF	SDNN, rMSSD, **pNN50**	HF, LF, LF/HF, TP	No

NCEP-ATP III: National Cholesterol Education Program’s Adult Treatment Panel III. IDF: International Diabetes Federation. AHA: American Heart Association. NHLBI: National Heart Lung and Blood Institute. Alberti et al., 2009 [33]. IC: Consensus definition from several national and international organizations. F/M: ratio female/male expressed in percentage (%).

**Table 3:** Long-term HRV modifications (time domain) produced by MS.

Reference	SDNN	SDANN	SDNNi	rMSSD	pNN50	pNN20	R-R
Stein et al., 2007 [21]	↓	↓					=
Rasic-Milutinovic et al., 2010 [27]	=			=			
Poliwczak et al., 2013 [24] ^M^	↓	↓	↓	↓	↓		
Wulsin et al., 2016 [23]	↓			↓			
Yoo et al., 2016 [32]	↓	↓	↓				
Balcioglu et al., 2016 [29]	↓	↓	↓	↓	↓		=
Ma et al., 2017 [11]	↓			=	=	↓	=
Slušnienė et al., 2019 [30]	=	=	↓	=	=		=
MacIorowska et al., 2020 [25]	↓			↓	↓		

↓: decreased in MS; =: without change; ^M^: only men.

**Table 4:** Long-term HRV modifications (frequency domain) produced by MS.

Reference	HF	LF	LF/HF	TP	VLF	ULF
Aso et al., 2006 [31]	=	↑	↑			
Stein et al., 2007 [21]				↓	=	↓
Gehi et al., 2009 [22]	↓	↓		↓	↓	↓
Assoumou et al., 2010 [26]	=	↓	↓	↓	↓	↓
Rasic-Milutinovic et al., 2010 [27]	↓	↓	↓	↓	↓	
Poliwczak et al., 2013 [24] ^M^	↓	↓	=	↓	↓	↓
Ma et al., 2017 [11]	↓	↓	=	=	↓	
Slušnienė et al., 2019 [30]	=	↓	↓		=	
Maciorowska et al., 2020 [25]	=	=	↑	=		

↓: decreased in MS; ↑: increased in MS; =: without change; ^M^: only men.

**Table 5:** Long-term HRV modifications (non-linear analysis) produced by MS.

Reference	SD1	SD2	α1	Multiscale Entropy
Stein et al., 2007 [21]			↓	
Assoumou et al., 2010 [26]			=	
Ma et al., 2017 [11]	=	↓		=
Slušnienė et al., 2019 [30]	=	=		

↓: decreased in MS; =: without change.

## Data Availability

The data presented in this study are available in the article.

## Appendix A

**Table A1:** Description of main time-domain HRV measurements reviewed.

Parameter	Description
SDNN	Standard deviation of the R-R interval series (overall variability).
SDANN	Standard deviation of the average R-R intervals for each 5 min segment of a 24 h HRV recording
SDNNi	Mean of the standard deviations of all the R-R intervals for each 5 min segment of a 24 h HRV recording
rMSSD	The root mean square of differences of successive R-R intervals.
pNN50	Successive R-R intervals that differ by more than 50 ms (expressed in percentage)
pNN20	Successive R-R intervals that differ by more than 20 ms (expressed in percentage)
R-R interval	Mean of successive R-R intervals.

**Table A2:** Description of main frequency-domain HRV measurements reviewed.

Parameter	Description
LF, HF, VLF, ULF	Ranges of the spectral components of the HRV
	LF: low frequency (0.04–0.15 Hz)
	HF: high frequency (0.15–0.4 Hz) VLF: very low frequency (0.0033–0.04 Hz) ULF: ultra-low frequency (<0.0033 Hz)
LF/HF	Ratio between LF and HF bands.
TP	Total power and includes all the bands together (<0.4 Hz).

**Table A3:** Description of main non-linear HRV measurements reviewed.

Parameter	Description
SD1	Standard deviation of the perpendicular point along the line of identity of the Poincaré plot. It represents the instantaneous beat-to-beat short-term variability.
SD2	Standard deviation of the perpendicular point along the line of identity of the Poincaré plot. It represents the instantaneous beat-to-beat long-term variability.
α1	Short terms fluctuations (4–12 beats) of detrended fluctuation analysis. The slopes of a log-log plot (correlation measure as a function of segment length).
Multiscale entropy	Measures the complexity of R-R time series, based on the analysis of sample entropy
